# A Zinc(II) Schiff Base Complex as Fluorescent Chemosensor for the Selective and Sensitive Detection of Copper(II) in Aqueous Solution

**DOI:** 10.3390/s23083925

**Published:** 2023-04-12

**Authors:** Ivan Pietro Oliveri, Agostino Attinà, Santo Di Bella

**Affiliations:** Dipartimento di Scienze Chimiche, Università di Catania, I-95125 Catania, Italy

**Keywords:** Zn(salen-type) complexes, fluorescent chemosensor, paper-based sensor strips, transmetalation, Cu^2+^ detection

## Abstract

The development of chemosensors able to detect analytes in a variety of sample matrices through a low-cost, fast, and direct approach is of current interest in food, health, industrial, and environmental fields. This contribution presents a simple approach for the selective and sensitive detection of Cu^2+^ ions in aqueous solution based on a transmetalation process of a fluorescent substituted Zn(salmal) complex. Transmetalation is accompanied by relevant optical absorption changes and quenching of the fluorescence emission, leading to high selectivity and sensitivity of the chemosensor, with the advantage of not requiring any sample pretreatment or pH adjustment. Competitive experiments demonstrate a high selectivity of the chemosensor towards Cu^2+^ with respect to the most common metal cations as potential interferents. A limit of detection down to 0.20 μM and a dynamic linear range up to 40 μM are achieved from fluorometric data. By exploiting the fluorescence quenching upon formation of the copper(II) complex, simple paper-based sensor strips, visible to naked eyes under UV light, are used for the rapid, qualitative, and quantitative in situ detection of Cu^2+^ ions in aqueous solution over a wide concentration range, up to 10.0 mM, in specific environments, such as in industrial wastewater, where higher concentrations of Cu^2+^ ions can occur.

## 1. Introduction

Fluorescent chemosensors are widely employed to detect ions and neutral species [1]. Due to their high sensitivity, they allow the detection of trace amounts of the analyte of interest. Metal cations [2,3,4,5,6,7,8,9,10,11,12,13], in particular the Cu^2+^ ion [2,3,4,5,6,7,8,9,10,11,12,13,14,15], are among the most investigated.

Copper plays an important role as essential trace element in various biological processes [16]. Both a deficiency or an excessive copper intake cause harmful health effects [17,18]. Copper is widely used in various manufactures as well as in agriculture. Sources for copper contamination include agriculture, industrial waste, mining, and photovoltaics; therefore, copper pollution represents an issue for environmental and human health [19,20].

Zn(salen)-type complexes are characterized by varied photophysical properties strongly related to the structure of the diimine bridge [21,22], substituents on the salicylidene rings, and non-coordinating or coordinating nature of the solvent [23,24,25]. Among them, colorful complexes derived from 2,3-diaminomaleonitrile, Zn(salmal), are the most interesting also for their fluorescent properties [26,27,28,29,30,31,32,33,34,35].

**Scheme 1:**
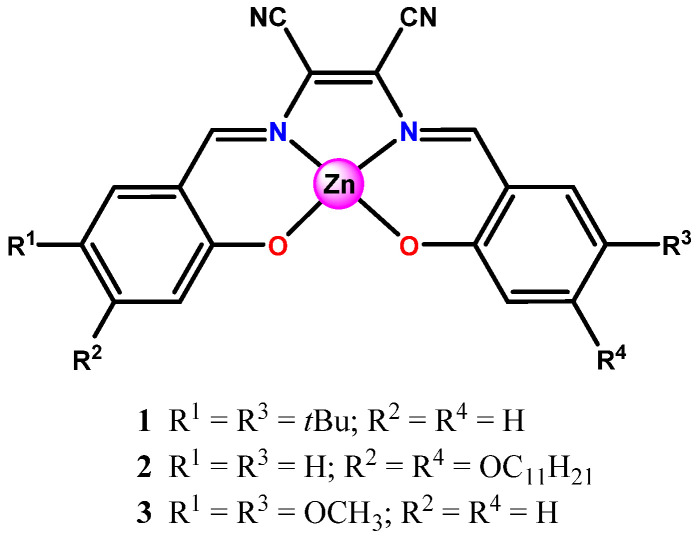
Structure of investigated substituted Zn(salmal) complexes.

We recently demonstrated that the Zn(salmal) complex (**1**, Scheme 1), substituted with 5,5′-*t*-butyl groups in the salicylidene rings to improve its solubility in a variety of solvents, in acetonitrile solution exhibits transmetalation with divalent ions of the first transition series [36]. Transmetalation is strongly influenced from the Lewis basicity of the solvent, i.e., from the stability of the starting **1** solvent adduct, and nature of the counteranion. In any case, transmetalation with Cu^2+^ ions is faster than that with the other cations. Therefore, complex **1** in acetonitrile solution is a useful colorimetric chemodosimeter for the selective detection of Cu^2+^ ions in aqueous solution [37]. As the formation of the paramagnetic Cu(salmal) complex upon transmetalation of 1 involves a quenching of the fluorescence, complex **1** has also been involved for the fluorometric detection of Cu^2+^. However, although the 5,5′-*t*-butyl substitution in Zn(salmal) enhances its solubility, on the other hand it leads to a very poor fluorescence, presumably for the specific substitution pattern in the 5,5′-positions of the salicylidene rings [38]. In fact, the resulting fluorometric sensitivity of **1** towards Cu^2+^ is slightly lower than that obtained from the colorimetric detection [37]. It is therefore expected that starting from a complex having a higher fluorescence, a parallel significantly higher sensitivity can be achieved. To this end, the Zn(salmal) complex (**2**, Scheme 1), substituted with 4,4′-alkoxy groups in the salicylidene rings, ensures a suitable solubility and a sizable fluorescence emission to be a good candidate for the fluorescent detection of Cu^2+^ ions in aqueous solution by transmetalation.

In this contribution we report a simple, straightforward approach for the selective and sensitive fluorescent detection of Cu^2+^ ions in aqueous solution by transmetalation, either in solution or as a test kit employing filter paper as a portable and displayable photonic device for the in situ detection. 

## 2. Materials and Methods

### 2.1. Materials and General Procedures

Complexes **1** and **2** were synthesized, purified, and characterized as previously reported [36,39]. All the chemicals used were purchased from Sigma-Aldrich (Merck, Darmstadt, Germany) and used as received. Zinc perchlorate hexahydrate was used for the template synthesis of complex **3**. *Caution: Perchlorate salts of metal compounds in the presence of organics are potentially explosive. Only small amounts of material should be cautiously handled.* Aqueous solutions of metal cations (as nitrate or perchlorate salts) were obtained by dissolving salts in distilled water and used as prepared. Stock solutions of complexes **1**–**3** in MeCN and THF were prepared by dissolving a known amount of the complex in the solvent using a volumetric flask. The solutions of complexes **1**–**3** used for spectrophotometric/spectrofluorometric measurements were prepared by diluting the stock solutions. The excitation wavelength for spectrofluorometric measurements was chosen in an isosbestic point. The fluorescence quantum yields for **3** in MeCN and THF were determined with respect to that of **2** in THF (*ϕ* = 0.24) [39]. Transmetalation experiments were performed as previously described [36]. The spectra for spectrophotometric and spectrofluorometric titrations were recorded after 10 min from the addition of Cu^2+^. 

### 2.2. Physical Measurements

ESI-MS spectra were recorded on a Thermo Fisher API 2000 mass spectrometer. ESI-MS spectra for complex **3** were recorded using MeOH/THF (2:1) solutions. ESI-MS spectra of complex **2** upon transmetalation with Cu^2+^ were achieved using MeCN solutions of **2** (40 μM) recorded immediately after the addition of 2 equiv. of an aqueous solution of Cu(NO_3_)_2_. ^1^H NMR, optical absorption, and fluorescence measurements were recorded with a Varian Unity S 500 (499.88 MHz for ^1^H) spectrometer, an Agilent Cary 60 spectrophotometer, and JASCO FP-8200 spectrofluorometer (JASCO Europe, Cremella, Italy), respectively.

### 2.3. Synthesis of Complex **3**

Complex **3** was synthesized using the same procedure adopted for the synthesis of complex **1** [36]. 2,3-diaminomaleonitrile (0.0541 g, 0.500 mmol) in methanol (10.0 mL), 5-methoxy-2-hydroxybenzaldehyde (125 µL, 1.00 mmol), zinc(II) perchlorate hexahydrate (0.223 g, 0.600 mmol), and triethylamine (0.500 mL). Dark-grey powder (0.198 g, 90%). ESI-MS: *m/z* = 439 [M+H^+^]. ^1^H NMR (500 MHz, DMSO-*d*_6_): *δ* = 8.58 (s, 2H, C*H* = N), 7.10 (dd, ^3^J_HH_ = 9.00 Hz, ^4^J_HH_ = 3.00 Hz, 2H; Ar*H*), 7.00 (d, ^3^J_HH_ = 9.00 Hz, 1H; Ar*H*), 6.74 (d, ^3^J_HH_ = 9.00 Hz, 2H; Ar*H*), and 3.71 (s, 6H, OC*H*_3_). 

### 2.4. Fabrication of Paper-Based Sensors and Sensing Experiments

Paper-based sensors (PBSs) were fabricated from filter paper, cut in 2.5 cm × 2.5 cm squares, using the dipping technique. Paper strips were dipped for 10 s into a THF solution of **2** (10 µM) and dried by solvent evaporation at room temperature in air. No change in the PBS performance was observed in paper strips prepared from solutions of **2** in MeCN; therefore, solutions of the more volatile THF solvent were used. Sensing experiments were performed by dipping PBS into aqueous solutions of various metal cations (as nitrate or perchlorate salts) at known concentration for 10 s and dried at room temperature for 30 min. Fluorescence changes were monitored under an UV lamp (λ_exc_ = 365 nm).

## 3. Results and Discussion

### 3.1. Photophysical Properties of Substituted Zn(salmal) Complexes

To find suitable fluorescent Zn(salmal) complexes for sensing metal cations via transmetalation, the new 5,5′-methoxy substituted complex **3** was synthesized and characterized, of which the photophysical properties in acetonitrile (MeCN) (Figure S1) and tetrahydrofuran (THF) (Figure S2) are compared with those of **1**. Although substitution of 5,5′-*t*-butyls with 5,5′-methoxy groups in the salicylidene rings of Zn(salmal) leads to an appreciable color change in the solutions, from violet (MeCN), blue-violet (THF) for **1** to indigo-blue for **3**, a very low fluorescence emission is observed for the latter complex (Table S1). Therefore, it can be inferred that the substitution pattern in 5,5′-positions of the salicylidene rings, and not the kind of the substituents (alkyl or alkoxy), is responsible for the low fluorescence emission of both complexes.

On the basis of these results, we decided to explore the complex **2** for the following transmetalation studies (Section 3.2). In both MeCN and THF solvents, solutions of **2** are characterized by an intense optical absorption band centered at λ_max_ = 542 nm in MeCN and λ_max_ = 551 nm in THF, responsible for their fuchsia color, and by a strong orange fluorescence emission, λ_max_ = 593 nm in MeCN (*ϕ* = 0.07) and λ_max_ = 594 nm in THF (*ϕ* = 0.24) [39]. 

### 3.2. Transmetalation Studies

Transmetalation, or metal exchange, is usually defined as the exchange of metal cations between an organometallic or coordination complex and a free metal cation, or metal exchange between two organometallic or coordination complexes [40]. This allows exploiting transmetalation to synthesize new molecular or supramolecular architectures and to develop new chemosensors and catalysts [12,41,42,43,44,45]. Despite his pivotal role in organometallic and coordination chemistry, transmetalation involving Zn(salen)-type complexes is almost unexplored [46,47,48]. 

Recently, we studied the transmetalation properties of the Zn(salmal) complex **1** in MeCN and N,N-dimethylformamide (DMF) solutions with divalent ions of the first transition series, in order to elucidate the role of stability of the (**1**·solvent) adduct and the nature of the counteranion on the transmetalation process [36]. 

In this work, the transmetalation properties of **2** towards some common divalent metal cations, in two solvents with a different Lewis basicity, were first explored in order to find the best way to use **2** as a fluorescent probe for the detection of metal ions in aqueous solution. In particular, the transmetalation properties of **2** in MeCN and THF were investigated. Both are coordinating solvents, but with different stability of the related **2**·MeCN and **2**·THF adducts [49]. Thus, the binding constant, *K*, of the **2**·MeCN adduct (log *K* = 1.32) is more than an order of magnitude lower than that of **2**·THF adduct (log *K* = 2.46) therefore, **2**·THF is thermodynamically more stable than **2**·MeCN, and this is expected to be reflected on the transmetalation properties of **2** in these two solvents. 

**Figure 1:**
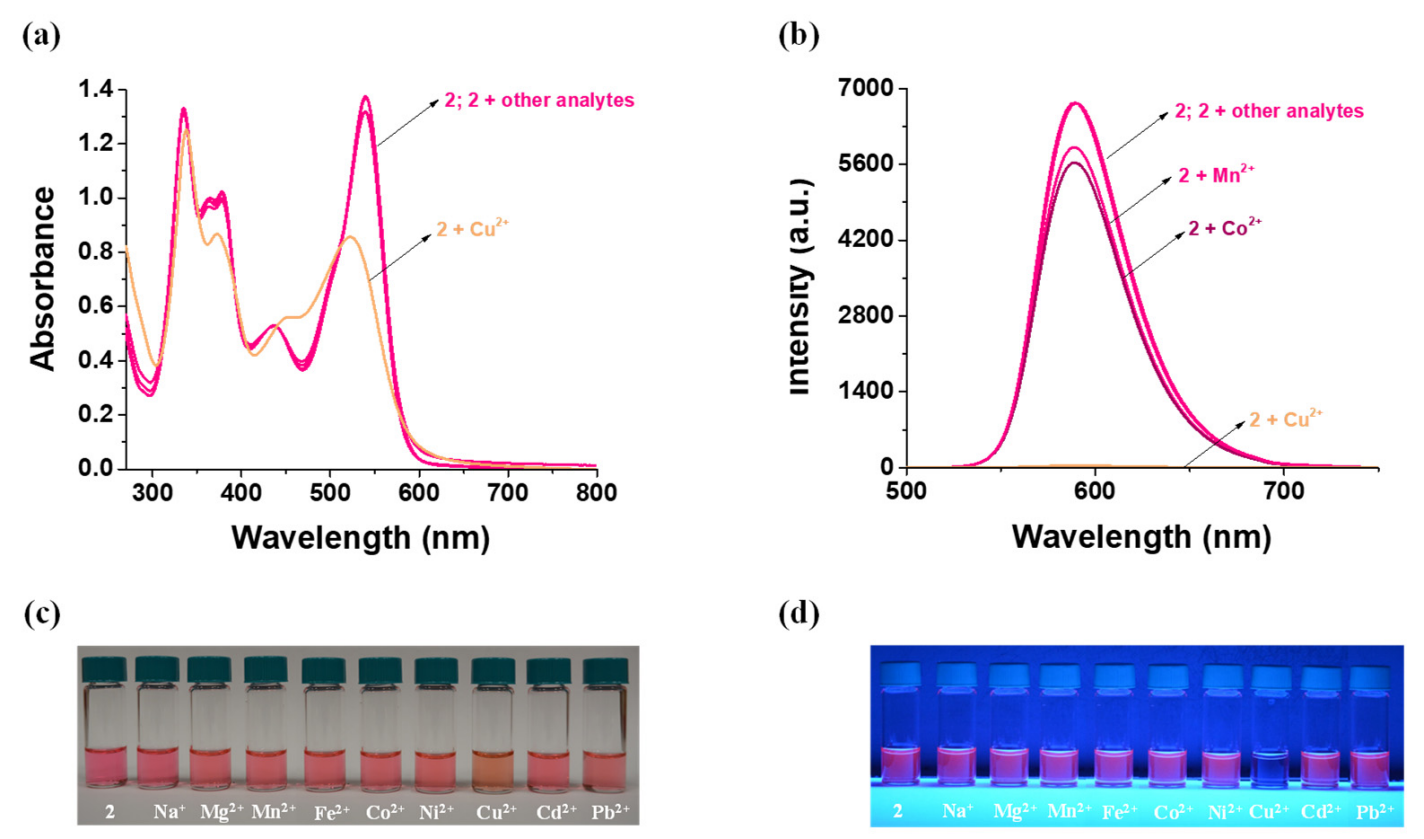
(**a**) UV-vis and (**b**) fluorescence (λ_exc_ = 421 nm) spectra of **2** (40 μM solution in MeCN) before and after the addition of 2.0 equiv. of various metal cations. Photographs under natural light (**c**) and under 365 nm light (**d**) after the addition of 2.0 equiv. of various metal cations.

The transmetalation properties of **2** in MeCN solution (40 µM) immediately after the addition of 2 equiv. of aqueous solutions of nitrate salts of Na^+^, Mg^2+^, Mn^2+^, Fe^2+^, Co^2+^, Ni^2+^, Cu^2+^, Cd^2+^, Hg^2+^, and Pb^2+^ are investigated through optical absorption and fluorescence measurements (Figure 1). Remarkable optical absorption changes are observed only in the case of the addition of Cu^2+^. In particular, the appearance of a new band centered at λ_max_ = 522 nm, having a shoulder at λ_max_ = 453 nm, is observed in the optical absorption spectrum, accompanied by a color change in the solution, from fuchsia to orange, and by a complete quenching of the fluorescence emission (Figure 1).

These optical absorption changes are analogous to those observed for **2** upon the addition of Cu^2+^ ions, and are consistent with a transmetalation process with formation of the paramagnetic substituted Cu(salmal) complex. This is corroborated by the fluorescence quenching, a distinctive phenomenon observed upon formation paramagnetic species [50] and by the presence of the protonated molecular ion in the ESI-mass spectrum (Figure S3). On the other hand, the addition of all the other cations involves small or negligible changes in the optical absorption and fluorescence spectra of **2**.

To monitor the transmetalation behavior of MeCN solutions of **2** upon the addition of 2 equiv. of the involved metal cations, the optical absorption and fluorescence spectra were recorded over time. Spectroscopic variations, consistent with a transmetalation, were observed only in the case of the addition of Mn^2+^, Co^2+^, and Ni^2+^ (Figure S4). In particular, after 30 h from the addition of Mn^2+^ and Co^2+^ ions, a hypochromism (and broadening only for Co^2+^) of the absorption band centered at λ_max_ = 522 nm were observed; whereas for the Ni^2+^ cation the appearance of a new band at λ_max_ = 508 nm, was found. Moreover, a turn-off of the fluorescence emission of **2** after 30 h from the addition of Co^2+^ and Ni^2+^ ions was found, while for the Mn^2+^ ion only a partial quenching of fluorescence was observed. Moreover, for the latter cation, no further fluorescence quenching was observed even after a longer time.

The transmetalation study of **2** in THF shows substantial differences with respect to the behavior observed in MeCN. In particular, no optical absorption changes and negligible fluorescence variations are found immediately after the addition of 2 equiv. of aqueous solutions of the metal cations sensitive to transmetalation (Mn^2+^, Co^2+^, Ni^2+^, and Cu^2+^) (Figure S5). After 30 h from the addition of the metal cations to THF solutions of **2**, only for Cu^2+^ a complete transmetalation is observed, as supported by the turn-off of the fluorescence (Figure S6). These results are consistent with the higher stability of the **2**·THF adduct with respect to **1**·MeCN adduct, which in turn, is reflected in a slower transmetalation process.

All these data together suggest that MeCN solutions of the complex **2** are suitable for the selective and sensitive fluorescent detection of Cu^2+^ ions in aqueous solution by transmetalation. 

### 3.3. Detection of Cu^2+^ Ions: Studies in Solution

**Scheme 2:**
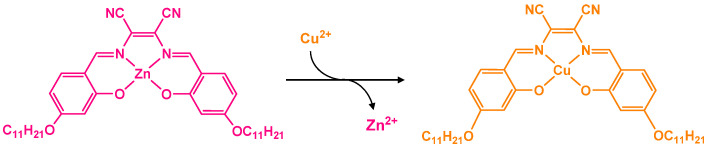
Mechanism of sensing of Cu^2+^ by transmetalation.

The remarkable optical absorption variations and the turn-off of the fluorescence emission of MeCN solutions of **2** upon the addition of aqueous solutions of Cu^2+^, as a consequence of the fast transmetalation of **2** with this metal cation respect to the others investigated, allow the selective and sensitive detection of Cu^2+^ ions in aqueous solution. The sensing mechanism is sketched in Scheme 2.

**Figure 2:**
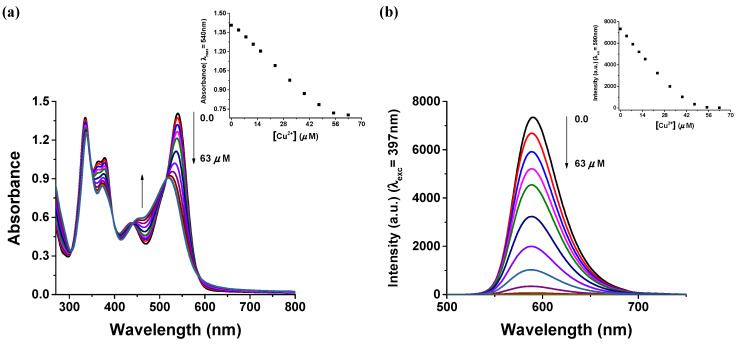
Optical absorption (**a**) and fluorescence (**b**) titration curves of **2** (40 μM solution in MeCN; λ_exc_ = 397 nm) with the addition an of aqueous solution of Cu(NO_3_)_2_. The concentration of Cu^2+^ added varied from 0 to 63 μM. Arrows indicate optical absorption and fluorescence changes upon addition of Cu^2+^. Insets: Variation in the absorbance at λ_max_ = 540 nm and fluorescence at λ_em_ = 590 nm as a function of the concentration of Cu^2+^ added.

In order to quantify the sensitivity of **2** towards Cu^2+^, in terms of binding constant and limit of detection (LOD), spectrophotometric and spectrofluorometric titrations were achieved (Figure 2).

The progressive addition of Cu^2+^ to MeCN solutions of **2** involves a parallel decreasing of the absorption band at 540 nm and the formation of a new band at 520 nm. These optical absorption variations and the presence of multiple isosbestic points are consistent with the transmetalation of **2** with formation of the substituted Cu(salmal) complex. This is corroborated by the progressive quenching of fluorescence upon the addition of Cu^2+^. The saturation was reached after the addition of 1.4 equiv. of Cu^2+^ and both binding isotherms, obtained using optical absorption and fluorescence data, show a wide linear dynamic range in the titration range 0–40 μM of the Cu^2+^ added (Figure 2). 

The binding constant for the formation of the Cu(salmal) complex upon transmetalation was achieved via Benesi–Hildebrand plots [51] (Figure S7). The linearity of the Benesi–Hildebrand plots is consistent with a 1:1 stoichiometry of the transmetalation process, as demonstrated by the Job’s plot analysis (Figure S8) [52,53].

The log *K* values for the transmetalation of complexes **1** and **2** with Cu^2+^ are comparable (Table S2) and this indicates a negligible influence of the substitution pattern in the positions of the salicylidene rings on the thermodynamics of the transmetalation process.

According to the IUPAC definition [54,55], the LOD of **2** towards Cu^2+^ was determined to be 0.20 μM using fluorometric data (Figure S9). This LOD value, lower than the limit of Cu^2+^ recommended by the World Health Organization (WHO) guidelines for water quality (30 μM) [56,57,58], is comparable to LOD values reported in the literature for efficient fluorescent chemosensors for detection of Cu^2+^ in aqueous solution [2,14,15], including more relevant recent contributions [59,60,61,62,63], with the advantage of not requiring any sample pretreatment or pH adjustment.

**Figure 3:**
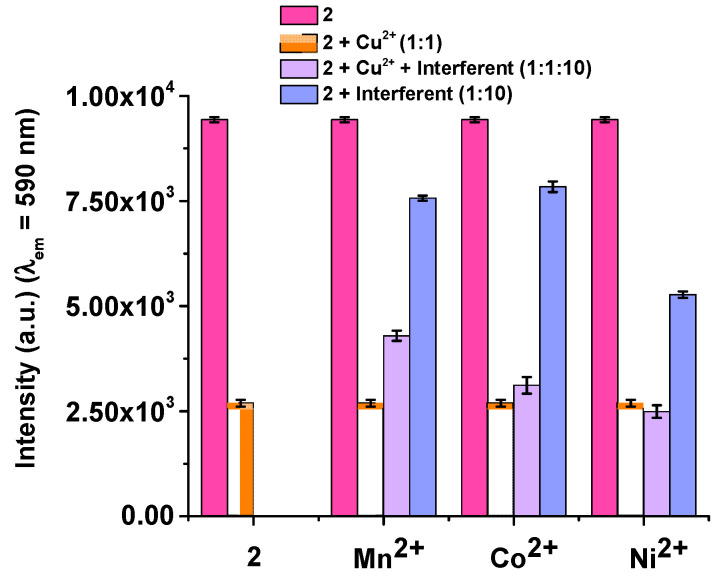
Fluorescence emission intensity of **2** at 590 nm (40 μM solution in MeCN, λ_exc_ = 397 nm; fuchsia bars); upon the addition of 1 equiv. of Cu^2+^ (light orange bars); upon the addition of 1 equiv. of Cu^2+^ in the presence of 10 equiv. of the interferent (lilac bars). The fluorescence emission intensity of **2** at 590 nm upon the addition of 10 equiv. of the interferent is represented with bright yellow bars.

To assess the selectivity of **2** towards Cu^2+^, competitive experiments were performed. In particular, the variation in the fluorescence of **2** upon the addition of 1 equiv. of Cu^2+^ in the presence of 10 equiv. of Mn^2+^, Co^2+^, and Ni^2+^ as potential interferents, was investigated (Figure 3).

The fluorescence variation of **2** upon the addition of 1 equiv. of Cu^2+^ is almost identical to that observed upon the addition of 1 equiv. of Cu^2+^ in the presence of 10 equiv. of the interferent metal cations. Although the addition of these metal cations to **2** (1:10 equiv. ratio) leads to some fluorescence variation, these changes remain much lower compared with those achieved in the presence of Cu^2+^ (1:1 equiv. ratio). The addition (up to 10^3^ equiv. excess) of the other above-investigated metal cations (Na^+^, Mg^2+^, Fe^2+^, Cd^2+^, Hg^2+^, and Pb^2+^, not included in Figure 3 for simplicity) does not affect either the fluorescence of **2** or the fluorescence variation of **2** upon the addition of 1 equiv. of Cu^2+^, therefore, these results highlight the remarkable high selectivity **2** towards Cu^2+^, even compared with that found for **1** [37].

### 3.4. Detection of Cu^2+^ Ions: Paper-Based Sensors

The very good sensing characteristics of **2** for Cu^2+^ ions prompted us to explore simple solutions for its application in disposable sensors. Paper is a low-cost substrate that can be useful for fabricating disposable and portable devices for rapid in situ detection of various analytes, including metal cations [64,65,66].

PBSs were fabricated by dipping paper strips in a THF solution of **2**. After drying, PBS strips show an orange fluorescence under the UV lamp (λ_exc_ = 365 nm) that can be seen by naked eyes (Figure S10). PBS strips were then dipped in an aqueous solution of Cu(NO_3_)_2_ (5.0 × 10^−3^ M) and dried at room temperature. After this treatment, a complete quenching of the fluorescence of PBS is observed (Figure S10). This result is in agreement with the transmetalation process, analogous to that observed in solution, with formation of the Cu(salmal) complex.

To investigate the selectivity of PBS towards Cu^2+^, PBS strips were dipped into solutions of the various cations (Na^+^, Mg^2+^, Mn^2+^, Fe^2+^, Co^2+^, Ni^2+^, Cu^2+^, Cd^2+^, and Pb^2+^) and air-dried at room temperature. In all cases, negligible or no change in the fluorescence emission is observed, except for the Cu^2+^ ions (Figure S11), demonstrating that the selectivity of **2** towards Cu^2+^ is also reflected in sensing experiments using PBS. Therefore, PBS can be used as disposable test strips for the rapid detection of Cu^2+^ in aqueous solutions with an on–off fluorescence mechanism.

**Figure 4:**
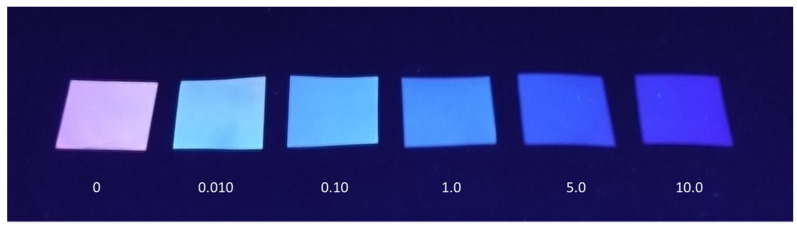
Photographs under 365 nm light of PBS strips after dipping in aqueous solution of different concentration of Cu^2+^ (range 0–10.0 × 10^−3^ M).

The quantitative detection of Cu^2+^ was also investigated by dipping PBS strips into aqueous solutions of different concentrations of Cu^2+^. As shown in Figure 4, on increasing the concentration of Cu^2+^ the fluorescence of PBS strips gradually turn-off, with a fluorescent color change from orange to dark blue, thus demonstrating that PBS strips can be used for the quantitative detection of Cu^2+^ ions in aqueous solution over a wide concentration range, up to 10.0 mM, compared with dynamic linear range found in solution. Therefore, PBS strips can be applied as a test for the detection of Cu^2+^ ions in aqueous solutions in specific environments, e.g., in industrial wastewater [19,67,68], where higher concentrations of Cu^2+^ ions can occur.

## 4. Conclusions

The availability of simple, low-cost, fast, and direct detection, without any treatment of the sample, is needed for real-time monitoring of analytes in specific environments. Here we present a straightforward approach for the selective and sensitive fluorescent detection of Cu^2+^ ions in aqueous solution, either in solution or using paper-based sensor strips as disposable tests for the in situ detection.

The sensing mechanism involves a fast transmetalation of a fluorescent substituted Zn(salmal) complex with Cu^2+^ ions, with formation of the paramagnetic substituted Cu(salmal) complex. The process is accompanied by relevant optical absorption changes and quenching of the fluorescence emission, leading to high selectivity and sensitivity, with the advantage of not requiring any sample pretreatment or pH adjustment. The fluorescence quenching upon formation of the copper(II) complex allowed the development of disposable paper-based sensor strips for the rapid, qualitative, and quantitative in situ detection of Cu^2+^ ions in aqueous solution over a wide concentration range, even in specific environments, for example in industrial wastewater, where higher concentrations of Cu^2+^ ions can occur.

## Data Availability

The data that support the findings of this study are available in the Supplementary Materials of this article.

## Supplementary Materials

The following supporting information can be downloaded at: https://www.mdpi.com/article/10.3390/s23083925/s1, Figure S1: Optical absorption and fluorescence emission spectra of complexes **1**–**3** in MeCN; Figure S2: Optical absorption and fluorescence emission spectra of complexes **1**–**3** in THF; Table S1: Absorption and emission data for complexes **1**–**3**; Figure S3: ESI-MS spectrum of **2** recorded after the addition of Cu^2+^; Figure S4: Optical absorption and fluorescence emission spectra of **2** in MeCN recorded after 30 h the addition of 2.0 equiv. of various metal cations; Figure S5: Optical absorption and fluorescence emission spectra of **2** in THF recorded after the addition of 2.0 equiv. of various metal cations; Figure S6: Optical absorption and fluorescence emission spectra of **2** in THF recorded after 30 h the addition of 2.0 equiv. of various metal cations; Figure S7: Benesi–Hildebrand plots from optical absorption and fluorescence emission data for the calculation of the binding constant of **2**; Figure S8: Job’s plot for the transmetalation of **2** with Cu^2+^ in MeCN; Table S2: Binding constants of complexes **1** and **2** for the transmetalation process with Cu^2+^; Figure S9: Linear best fit for the fluorometric titration of **2** in MeCN as a function of the concentration of Cu^2+^ added for the determination of LOD; Figure S10: Photographs under natural and UV light of PBS strips before and after dipping in an aqueous solution of Cu^2+^; Figure S11: Photographs under 365 nm light of PBS strips after dipping in aqueous solutions of various metal cations.
