# Supplementary material for: A Zinc(II) Schiff Base Complex as Fluorescent Chemosensor for the Selective and Sensitive Detection of Copper(II) in Aqueous Solution
Source: Sensors (Basel). 2023 Apr 12;23(8):3925. doi: 10.3390/s23083925 (PMC10141078; doi:10.3390/s23083925)
Supplement: Supplementary file 1 [file sensors-23-03925-s001.zip › sensors-2305841-supplementary.pdf]

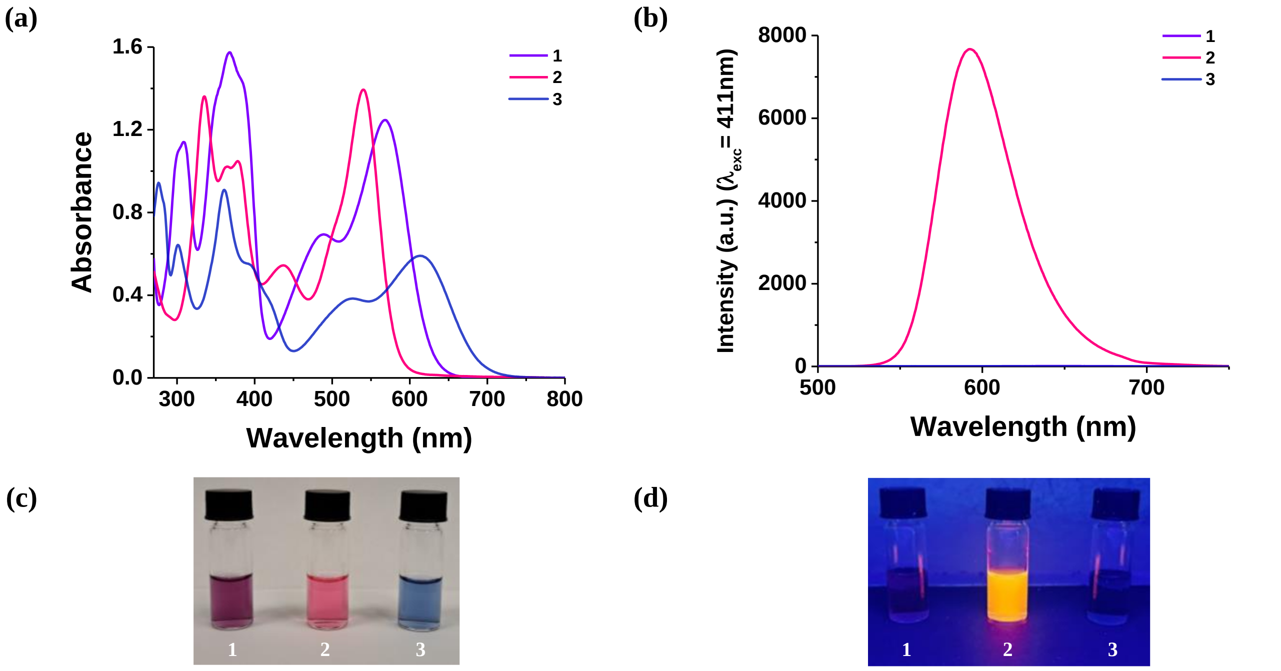

Figure S1. (**a**) Optical absorption and (**b**) fluorescence emission (λ_{exc} = 411 nm) spectra of complexes **1-3** (40 μM solution in MeCN). Photographs under natural light (**c**) and under 365 nm light (**d**).

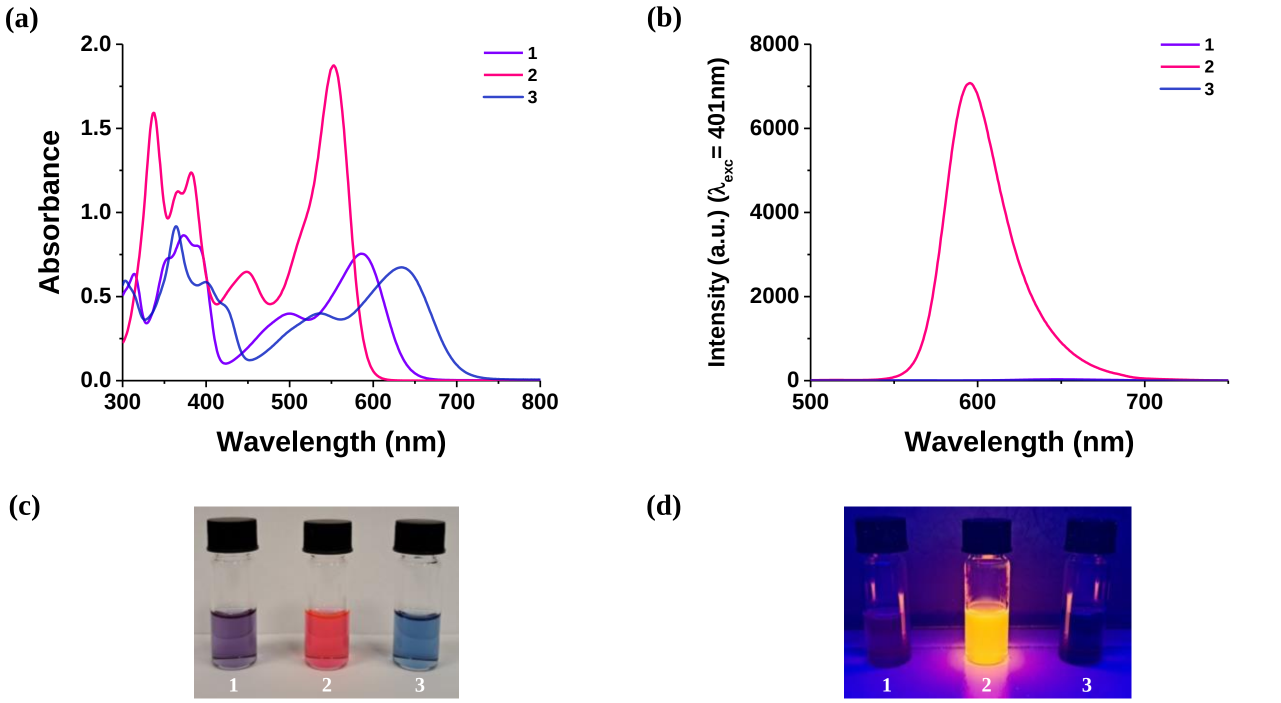

Figure S2. (**a**) Optical absorption and (**b**) fluorescence emission (λ_{exc} = 401 nm) spectra of complexes **1-3** (40 μM solution in THF). Photographs under natural light (**c**) and under 365 nm light (**d**).

Table S1. Absorption and emission data for complexes **1**-**3** (40 μM in MeCN and THF).

		Absorption		Emission	
complex	solvent	λ_{max} (nm)	Color	λ_{max} (nm)	Φ
**1**	MeCN	568 (487)	violet	-	< 0.01[a]
**1**	THF	586 (499)	blue-violet	-	< 0.01[a]
**2**	MeCN	542	fuchsia	593	0.07[b]
**2**	THF	551	fuchsia	594	0.24[b]
**3**	MeCN	614 (527)	indigo-blue	-	< 0.01
**3**	THF	634 (535)	indigo-blue	-	< 0.01

[a] From reference [37] in the main text; [b] From reference [39] in the main text.

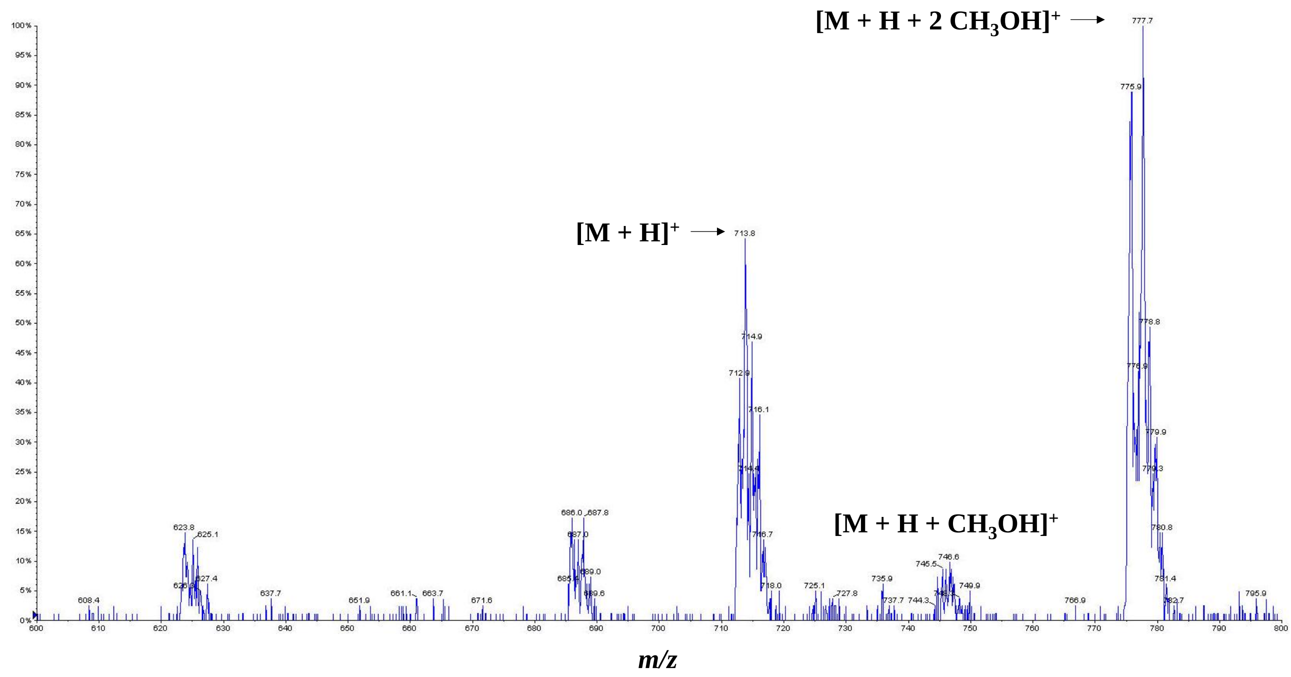

Figure S3. ESI-MS spectrum of MeCN solutions of **2** recorded immediately after the addition of 2 equiv. of an aqueous solution of $Cu(NO_3)_2$.

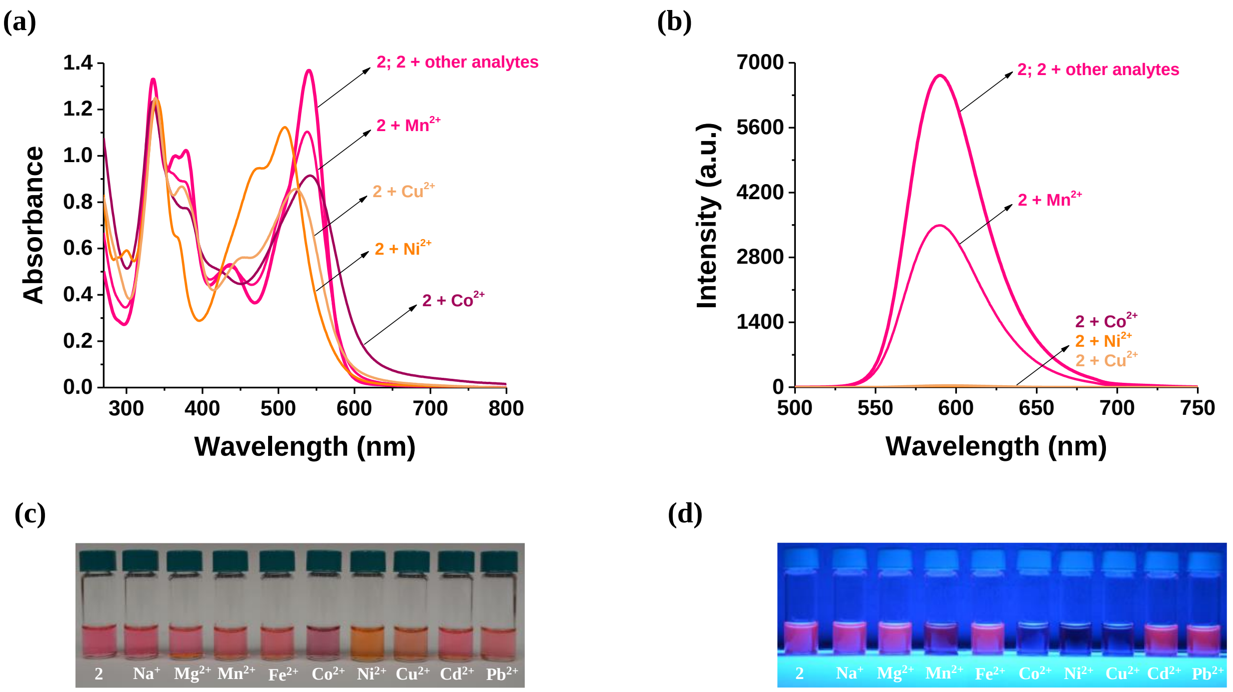

Figure S4. (**a**) Optical absorption and (**b**) fluorescence emission (λ_{exc} = 421 nm) spectra of **2** (40 μM solution in MeCN) and after the addition of 2.0 equiv. of various metal cations (as aqueous solutions of nitrate or perchlorate salts) recorded after 30 hours. Photographs under natural light (**c**) and under 365 nm light (**d**) after the addition of 2.0 equiv. of various metal cations recorded after 30 hours.

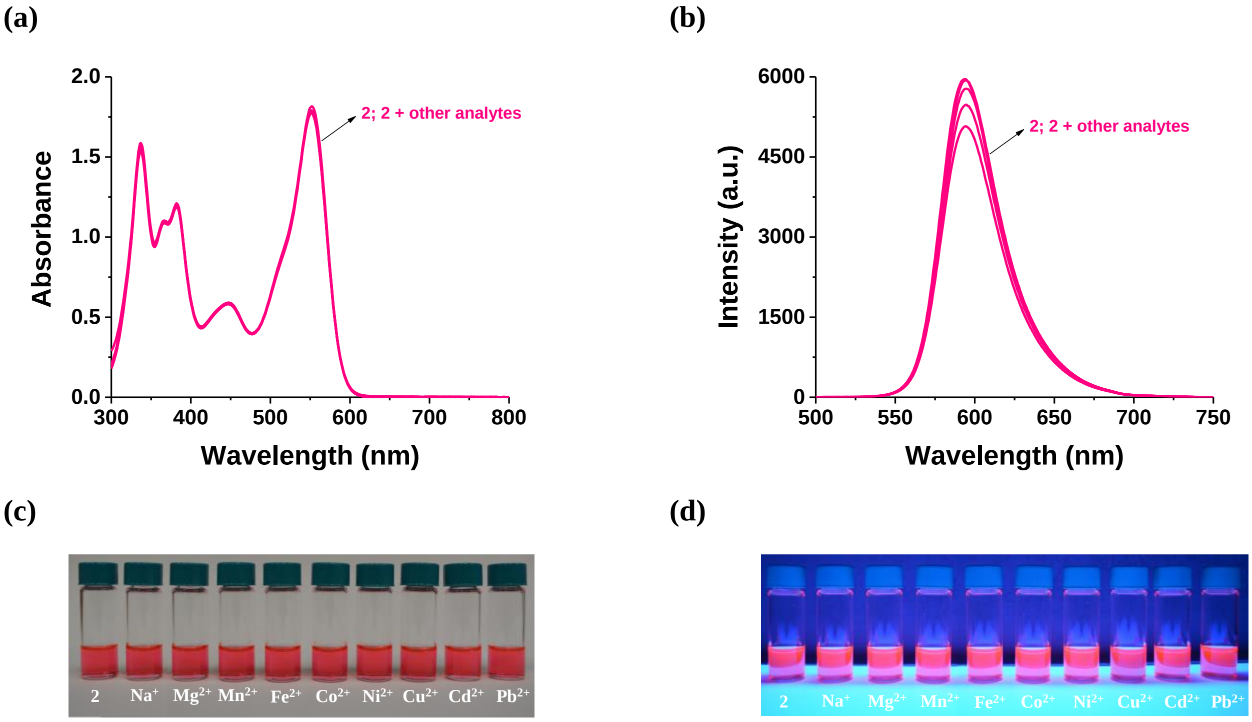

Figure S5. (**a**) Optical absorption and (**b**) fluorescence emission (λ_{exc} = 408 nm) spectra of **2** (40 μM solution in THF) and after the addition of 2.0 equiv. of various metal cations (as aqueous solutions of nitrate or perchlorate salts). Photographs under natural light (**c**) and under 365 nm light (**d**) after the addition of 2.0 equiv. of various metal cations.

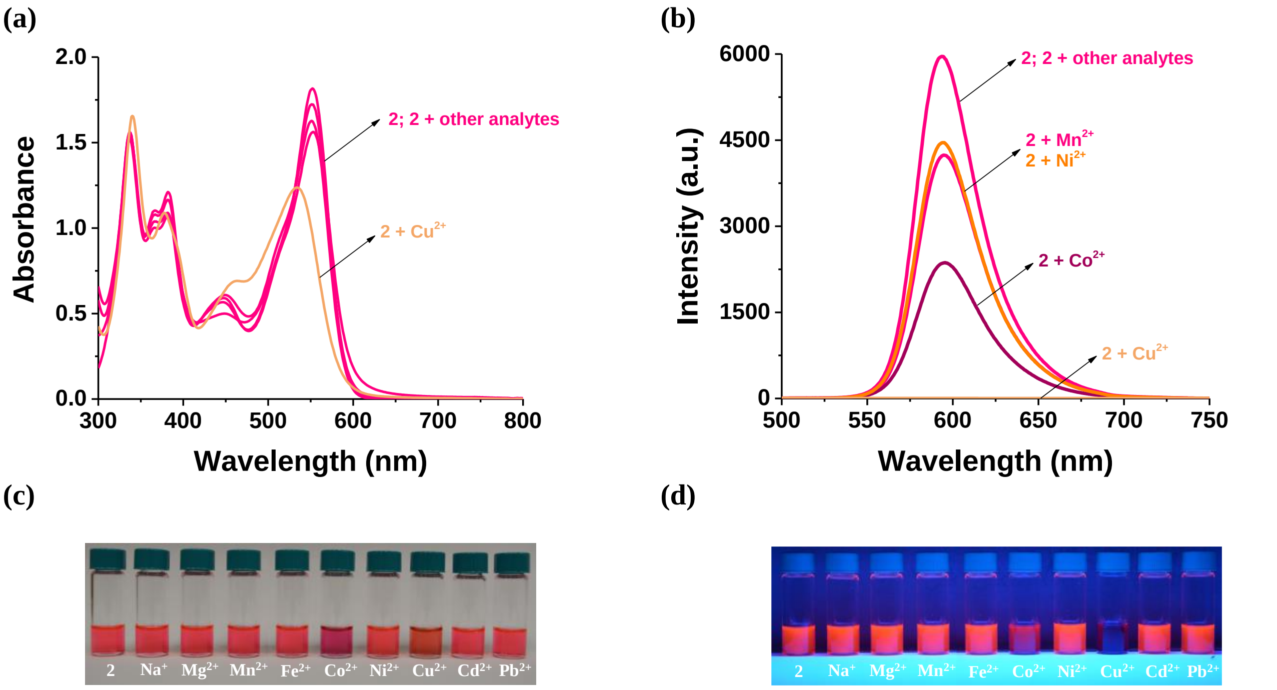

Figure S6. (**a**) Optical absorption and (**b**) fluorescence emission (λ_{exc} = 408 nm) spectra of **2** (40 μM solution in THF) and after the addition of 2.0 equiv. of various metal cations (as aqueous solutions of nitrate or perchlorate salts) recorded after 30 hours. Photographs under natural light (**c**) and under 365 nm light (**d**) after the addition of 2.0 equiv. of various metal cations recorded after 30 hours.

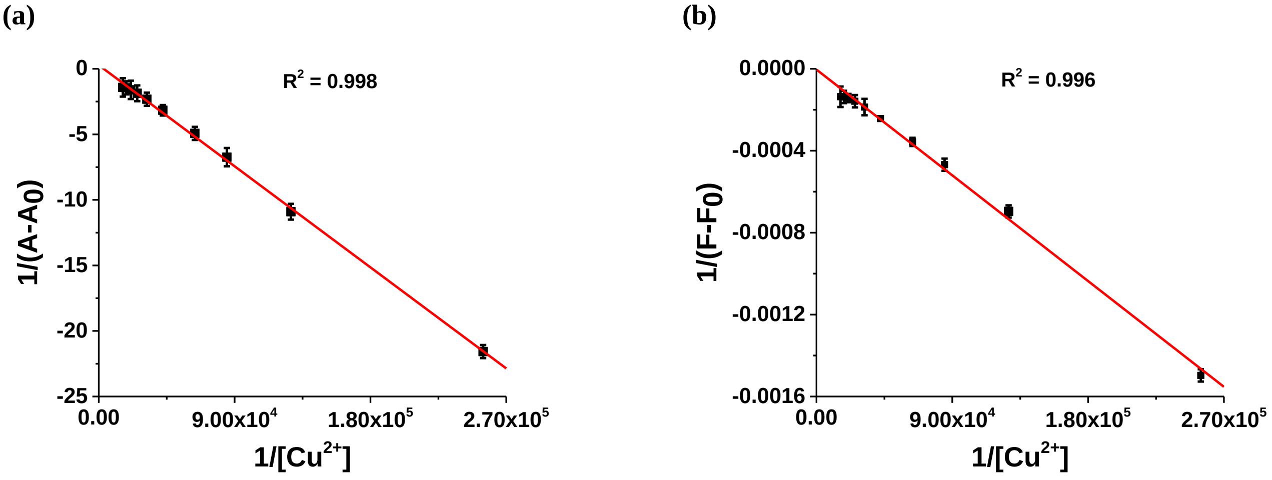

Figure S7. Benesi–Hildebrand plots obtained from optical absorption (λ_{max} = 540 nm) **(a)** and fluorescence emission (λ_{em} =590 nm) **(b)** for the calculation of the binding constant of **2** (40 μM solution in MeCN) with Cu^{2+}.

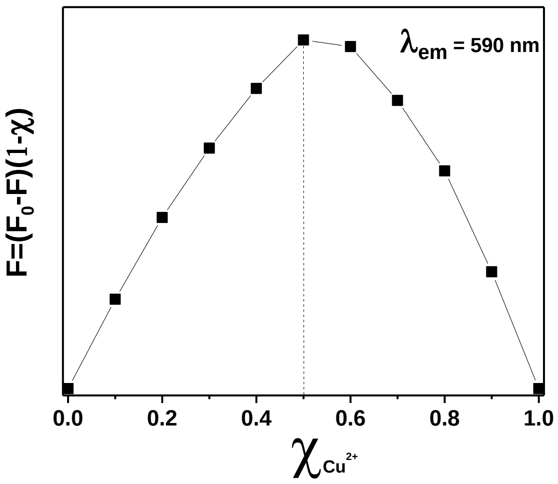

Figure S8. Job plot for the transmetalation of **2** with Cu^{2+} in MeCN (λ_{em} =590 nm). The total concentration of **2** and Cu^{2+} is 40 μM.

Table S2. Binding constants, log *K*, of complexes **1** and **2** for the transmetalation process with Cu^{2+} achieved by Benesi-Hildebrand plots.

	**log *K*** [a]	**log *K*** [b]
**1** [c]	3.8	3.4
**2**	3.5	3.3

[a] From absorption data; [b] From fluorescence data; [c] From reference [37] in the main text.

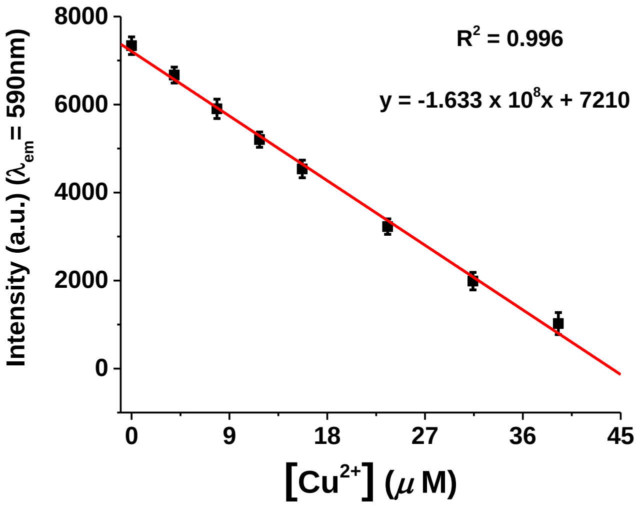

Figure S9. Linear best fit in the linear dynamic range for the titration of the 40 μM solution of **2** in MeCN (fluorescence intensity at λ_{em} = 590 nm as a function of the concentration of Cu^{2+} added) for the determination of LOD. LOD = 3σ/k where σ is the standard deviation of the blank (σ = 10 considering fifteen blank replicates), and k is the slope of the calibration line from the spectrofluorometric titration data.

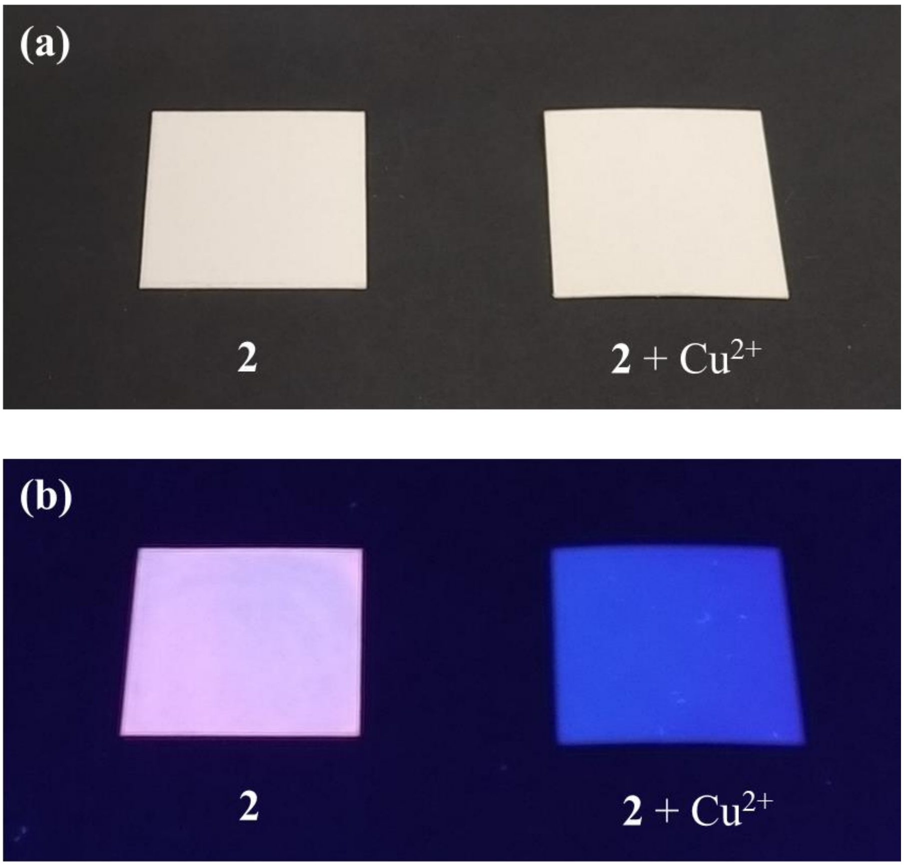

Figure S10. Photographs under natural light (**a**) and under 365 nm light (**b**) of PBS strips before (left) and after (right) dipping in an aqueous solution of Cu^{2+} (5.0×10^{-3} M).

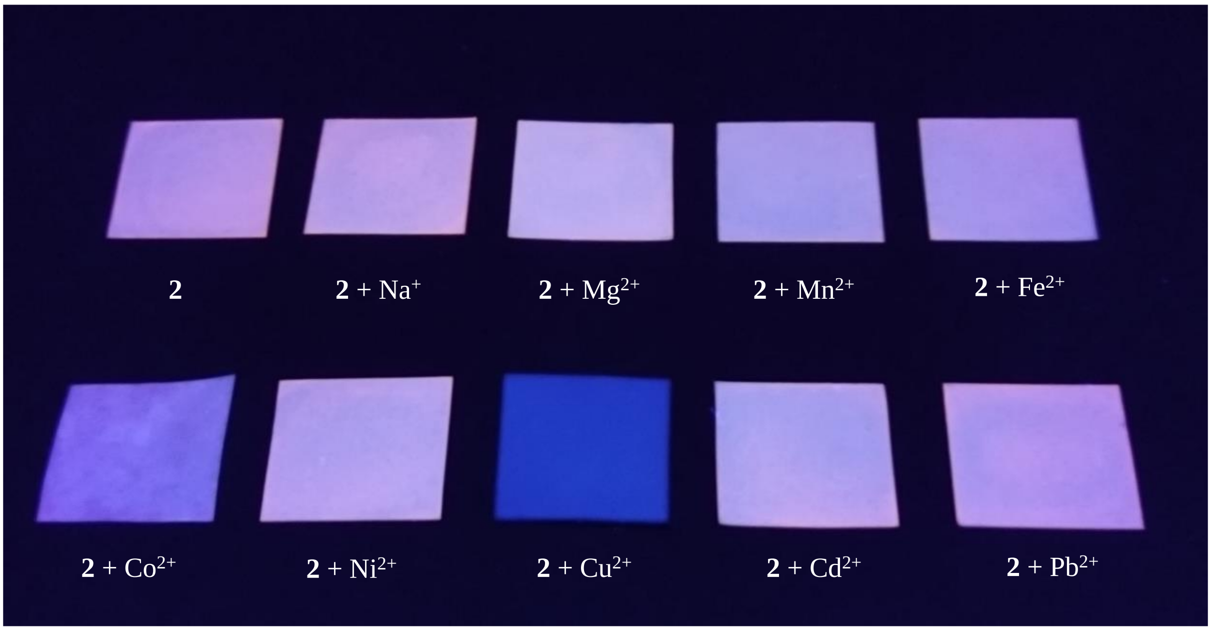

Figure S11. Photographs under 365 nm light of PBS strips before and after dipping in aqueous solutions (5.0×10^{-3} M) of the metal cations (as nitrate or perchlorate salts).
